# Strawberry Puree Functionalized with Natural Hydroxytyrosol: Effects on Vitamin C and Antioxidant Activity

**DOI:** 10.3390/molecules25245829

**Published:** 2020-12-10

**Authors:** Alejandra Bermúdez-Oria, Yougourthane Bouchal, África Fernández-Prior, Blanca Vioque, Juan Fernández-Bolaños

**Affiliations:** Department of Food Phytochemistry, Instituto de la Grasa (Spanish National Research Council, CSIC), Ctra. de Utrera km. 1, Pablo de Olavide University Campus, Building 46, 41013 Seville, Spain; aleberori@ig.csic.es (A.B.-O.); bouchalyougourthane@gmail.com (Y.B.); africafprior@hotmail.com (Á.F.-P.); bvioque@ig.csic.es (B.V.)

**Keywords:** antioxidant activity, functionalized, hydroxytyrosol, strawberry puree, vitamin C

## Abstract

The natural antioxidant hydroxytyrosol (HT) was used to functionalize a strawberry puree. The effect of the antioxidant on the stability of the two bioactive forms of vitamin C (ascorbic acid-AA and dehydroascorbic acid-DHAA) in strawberry puree stored at 4 °C, compared with the effect on a model system of AA in water, was investigated. In the absence of HT, the concentration of vitamin C in strawberry puree decreased but not in the model system. Low concentrations of HT in strawberry puree (0.05 and 0.1 mg HT/g puree) stabilized vitamin C and improved its antioxidant activity. However, at high concentrations of HT (from 0.5 mg HT/g puree), although the antioxidant activity improved, degradation of vitamin C occurred. Therefore, the concentration of HT used to obtain a functionalized strawberry puree it is very important. An adequate concentration increases the antioxidant activity and protects vitamin C from degradation, developing a functional food. However, an inadequate concentration of HT affects the vitamin C content, which is essential for the human diet because it cannot be biosynthetized by the organism.

## 1. Introduction

The importance of the consumption of fruits and vegetables to human health, due to their high content of antioxidant, is well known. Antioxidants reduce oxidative stress and are associated with a lower incidence of infections, cardiovascular diseases and cancers [1,2]. Strawberry fruit is a rich source of antioxidant compounds such as ascorbic acid (AA), with an extremely high content of phenols [3]. Ascorbic acid, and its oxidized form, dehydroascorbic acid (DHAA), are the bioactive forms of vitamin C, which are well known for their strong antioxidant activity as free radical scavengers, preventing the generation of ROS (reactive oxygen substances) [4]. The biochemical role of ascorbic acid is related to its ability to act as an electron donor or reducing agent, and participate in a wide range of essential metabolic reactions [5]. It is an essential vitamin, which is highly unstable, sensitive to oxygen and temperature and must be ingested because it cannot be synthetized by human metabolism. It is vital for the growth and maintenance of healthy bones, teeth, gums and blood vessels [6]. The phenolic compounds present in the strawberry fruit, and their biological properties, have been well described in previous works [7,8]. The strawberry contains high levels of flavonoids (flavonols, flavanols, anthocyanins and ellegitannin derivatives) that present a great number of beneficial health activities which protect against cardiovascular diseases and exhibit antiproliferative and anti-inflammatory effects [3]. The possible distribution of antioxidant activity in these metabolites in strawberry extracts was studied by Tulipani et al., (2008) [9], who indicated that vitamin C was responsible for more than 30%, followed by anthocyanins contributing to 25 to 40%; and the rest was composed mainly of ellagitannin derivatives and flavanols.

The phenolic antioxidants present in fruit juice protect the vitamin C content from oxidative degradation but depend on the class of polyphenols. The phenylpropanoids are less effective at conserving ascorbate than the polyphenolic flavonoids from blackcurrant juice [10,11]. Flavonol aglycones were good protectors of vitamin C, while glycosides were less effective [12], and anthocyanin showed antioxidant activity only in the presence of copper ions [13]. 

On the other hand, studies on strawberry have suggested that the bioactive forms of vitamin C exert a possible protective effect on the content of ellagic acid [14]. Ascorbic acid protects anthocyanins, but not quercitin, from oxidative degradation in elderberry [15] and stabilizes tea catechin at neutral and alkaline pH [16]. This protective effect of vitamin C on polyphenols appears to be due to the reduction of the oxidized form of polyphenols. This property has been used for the enzymatic synthesis of antioxidant hydroxytyrosol using tyrosol as a precursor. The enzyme tyrosinase catalyzes the reaction of tyrosol to hydroxytyrosol, and also the oxidation of this to o-quinone, which is reduced to hydroxytyrosol by vitamin C, which, in turn, is oxidized [17].

In this work, the addition of an exogenous antioxidant such as hydroxytyrosol, an important natural antioxidant recovered from olive oil waste, to strawberry puree was investigated in relation to its interaction with vitamin C. Hydroxytyrosol is a good candidate since it is considered one of most potent antioxidants present in olive fruit and virgin olive oil, with the strongest ROS scavenging properties in vivo and in vitro [18]. Its regular consumption was found to be beneficial for cardiovascular diseases, diabetes, cancer and neurodegenerative diseases [19]. In addition to this inherent multitude of beneficial health effects, it is possible to provide effective protection against the oxidation of vitamin C by the inhibition or delay of the implicated reaction. Although enzymatically synthesized hydroxytyrosol has been used to functionalize tomato juice, with a significant increase in antioxidant activity, the effect on the stability of vitamin C was completely lacking [20]. The aim of the present study was the use of a natural hydroxytyrosol extract to enrich and fortify strawberry puree and to evaluate its influence on the stability of vitamin C and total antioxidant activity. In this study, the synergistic, additive or antagonic antioxidant effect of the interaction between hydroxytyrosol and strawberry puree on in vitro antioxidant activity was investigated during storage using standard antioxidant assays (2,2-diphenyl-1-picrylhydrazyl (DPPH) radical, and ferric-reducing antioxidant power (FRAP)). The interactions between hydroxytyrosol and strawberry puree in relation to antioxidant activity were evaluated using the Compusyn program by combination indexes.

## 2. Results and Discussion

### 2.1. Evolution of Vitamin C and Hydroxytyrosol in a Model (Ascorbic Acid) Solution and in Strawberry Puree

**Figure 1:**
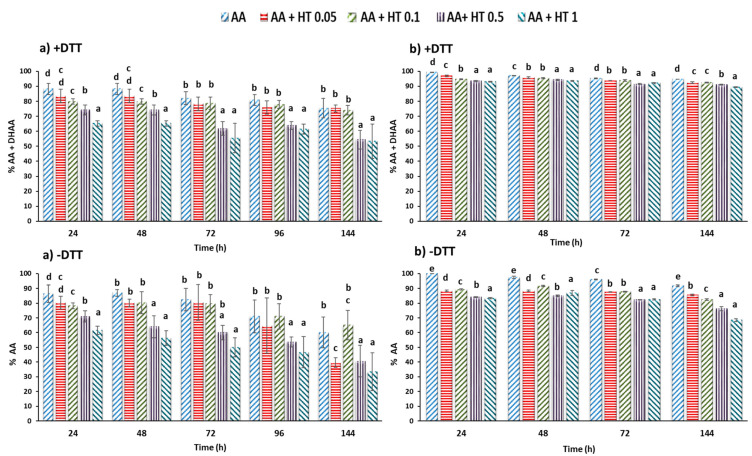
The evolution of the vitamin C content of a model system solution (0.6 mg/mL) with different concentrations of HT added (0.05, 0.1, 0.5 and 1 mg/mL of solution) stored at room temperature (**a**) and at 4 °C (**b**) with DTT (+DTT = total ascorbic [AA + DHAA]) and without DTT (−DTT only acid ascorbic, AA). The data are shown as mean ± SD of quadruplicates. Letters (a–e) indicate significant differences among groups at *p* < 0.05.

Figure 1 shows the evolution in vitamin C in a model solution control of ascorbic acid (AA 0.6 mg/mL) in an aqueous medium and in a model solution containing HT at four concentrations for 144 h (six days) of storage at room temperature (RT) and at 4 °C. Higher temperature accelerated vitamin C degradation, while at 4 °C the control retained almost 94.6% of the vitamin C. At room temperature (RT) it retained only 76.9% at 144 h. The added HT did not protect the vitamin C throughout storage for the two temperatures assayed. The rate of vitamin C (AA + DHAA) reduction at 1 mg/mL of HT was 10% and 42% after six days of storage at 4 °C and RT, respectively. The vitamin C was affected by all the HT concentrations (*p* ˂ 0.05) and by the temperature and storage time. On the other hand, the rate of degradation of added HT during storage at RT was relatively low (Table 1a) with a reduction of around 10–15%. These finding do not support the hypothesis that HT protects vitamin C from oxidative degradation or that HT may be protected by vitamin C, probably because only a certain reduction in the oxidized form of HT by AA, which is in turn oxidized, occurred. This is an oxidation-reduction system in which there is a H-atom or electron transfer. From the results obtained here and those reported by others [21], it was apparent that HT, with dihydroxy functionality and with a potent singlet oxygen (^·^O_2_) quencher [20], has greater in vitro antioxidant capacity than AA.

**Figure 2:**
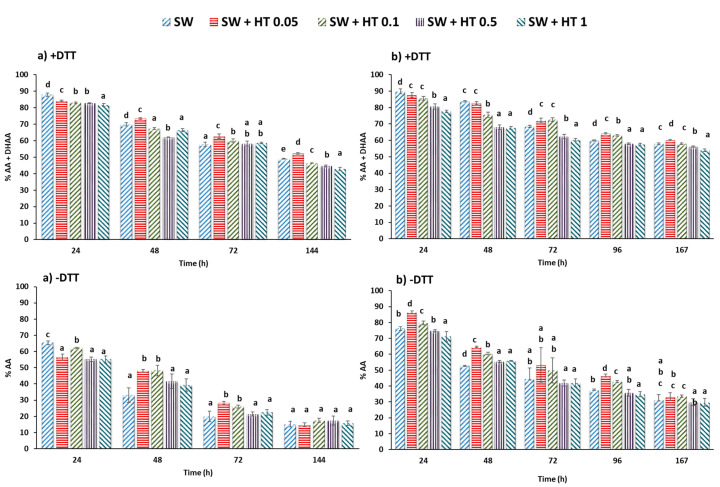
Evolution of vitamin C in strawberry (SW) puree of the Calinda varieties with different concentrations of HT added (0.05, 0.1, 0.5 and 1 mg/g strawberry) (**a**) and in the Inspire variety (**b**) with DTT (+DTT = total ascorbic [AA + ADHA]) and without DTT (−DTT only acid ascorbic, AA) during storage at 4 °C. Influence of HT added to different concentrations. The data are shown as mean ± SD of quadruplicates. Letters (a–d), indicate different differences between groups with *p* < 0.05.

The effectiveness of HT in a real food such as strawberry puree was also measured. The strawberry fruits used in the two experiments, from two different varieties, had a similar vitamin C (AA + DHAA) content with 63 and 58.3 mg/100 mL (100 mg). DHAA values were 6.3 and 7.5 mg/100 mL (100 mg) that represented about 10 and 12.9% of the vitamin C content in strawberry (Figure 2). These amounts of DHAA present in strawberry coincide with those reported in the literature [14,22]. In this case, storing at 4 °C showed in the control (without HT addition) a quicker reduction in vitamin C (AA + DHAA), with a degradation of 51 and 36% for the Calinda variety and 50% and 57% for the Inspire variety after 144 and 167 h of storage (Figure 2a,b +DTT). These results contrast with those previously obtained for the standard vitamin C solution stored at 4 °C (Figure 1b). This reduction could be attributable to the high content of polyphenols of the strawberry, which include free ellagic acid and polymeric ellagitannins among others, with pronounced antioxidant activity [3]. This could be in accordance with studies that suggested that the AA may have protective effects on ellagic acid oxidation in strawberries [14,23]. In the first experiment (Figure 2a +DTT), samples containing 0.05 mg/mL of HT had significantly higher vitamin C (AA + DHAA) content during 48, 72 or 144 h of storage than the control by 5, 8 or 6% (*p* ˂ 0.05). In the second experiment (Figure 2b +DTT), samples containing 0.05 and 0.1 mg/mL also presented significantly higher vitamin C content than the control in a range of 4.8 to 6.6% (*p* ˂ 0.05) after 72 h of storage. In both experiments, this effect was more evident, even with higher HT concentration when AA was quantified only (Figure 2a,b−DTT). 

**Figure 3:**
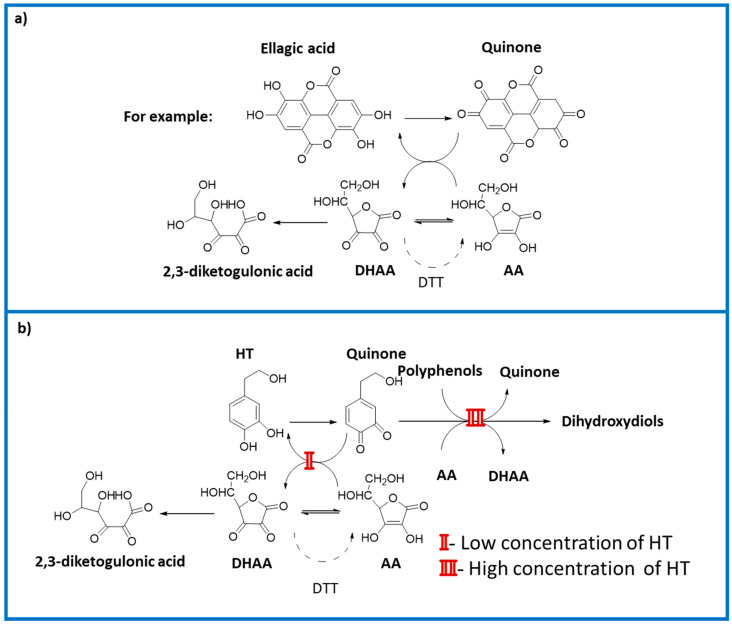
Summary scheme of the changes that take place in vitamin C in strawberry puree stored at 4 °C, without added HT (**a**), and in the presence of HT (**b**).

The addition of HT solutions at the low concentrations of 0.05 or 0.05 and 0.1 mg/mL, preserved vitamin C after 48 or 72 h of storage (Figure 2). Our findings, and the knowledge that vitamin C is a protector of ellagic acid in strawberries [14] and of polyphenols of plants in general, which are easily oxidized, suggest that depending of its concentration, HT prevents vitamin C from acting as a protector of the polyphenols in strawberry puree during storage, thus confirming the results of other authors [10,12]. In Figure 3a the changes in the AA in the strawberry control (without HT added) are represented and explain the reduction in the vitamin C (AA + DHAA) content in different solutions of control AA, which practically did not change during storage at 4 °C. In this case, the AA acted as a protector of the phenols in strawberry puree. Figure 3b presents two possibilities depending on the concentration of added HT. With lower HT concentration (I), the HT was oxidized to its catechol quinone during redox cycling and the AA of strawberry puree was able to reduce the o-quinone, thus regenerating the HT in the medium. Under these assay conditions, the HT content did not decrease, while vitamin C (AA + DHAA) decreased, but only slightly above the control strawberry (Table 1b). Conversely, in the presence of a high concentration of HT, from 0.5 mg/mL (II), the AA in strawberries was unable to reduce all the o-quinone formed from HT, with decreases in HT and AA concentrations. These primary oxidation products, o-quinones, are very unstable and highly reactive [24,25], and thus are likely to react further with other phenolic or nonphenolic compounds including the polyphenols and AA in strawberries. The loss in AA was accompanied by a loss in HT (Figure 2). These losses may be attributed to oxidation, but also in part to the interference of HT in the chain reaction, although its effectiveness depends on the concentration used. 

### 2.2. Antioxidant Activity of Functionalized Strawberry Puree

**Figure 4:**
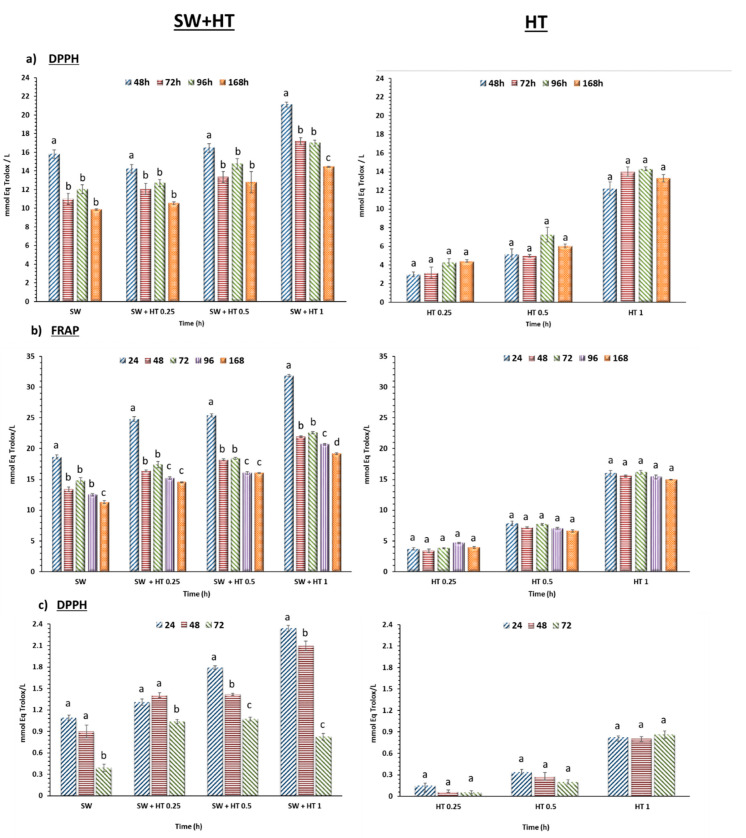
Antioxidant activity (mmol Eq Trolox/L) in the aqueous fraction obtained from the mixture of strawberry puree and hydroxytyrosol (SW+ HT) at 0.25, 0.5 and 1 mg/g fresh strawberry, and comparison with the activity of strawberry puree without added HT and the HT solution in an amount equivalent to that present in the mixture by the DPPH (**a**), and FRAP (**b**) and DPPH of the mixture of strawberry puree and hydroxytyrosol (**c**). The DPPH method was used, in which the strawberry puree (100 mg) was suspended in 7 mL of the DPPH reagent (3.8 mg/50 mL methanol). The data are shown as mean ± SD of quadruplicates. Letters (a–d) indicate significant differences among groups at *p* < 0.05. SW (Strawberry).

The antioxidant capacity of strawberry puree depends not only on its high content of vitamin C but also on the presence of polyphenols. The contribution of HT to the antioxidant potential of strawberry puree was determined using standard in vitro antioxidant assays (DPPH, and FRAP). In these experiments, HT was added to the strawberry puree to investigate three different final concentrations of 0.25, 0.50 and 1.00 mg/g of fresh strawberry. The potential interactions of the added HT with the different strawberry puree components were determined and compared to a strawberry puree control without HT. Strawberry puree was extracted using water and methanol:water (70:30). The content of phenols obtained by water was higher than in the methanol extract, thus water was selected as the extract to determine antioxidant activity. Appropriate dilutions in a linear range were used to calculate the antioxidant activity, which was expressed as mmol Eq.Trolox/L. The results demonstrated that the antioxidant effect depended on the relative amounts of the two components, HT and strawberry puree, in the mixture, and their evolution during storage. The results obtained experimentally for the mixture with different ratios were compared to the effects of the individual components analyzed separately, HT and strawberry puree extract. Figure 4a,b shows the results obtained by the methods DPPH and FRAP, respectively. The levels of antioxidant activity and their evolution with storage time were very similar for both procedures (DPPH and FRAP). The results show a clear decrease in activities with storage time, with the levels in the mixtures being considerably lower than the sum of the components analyzed individually. This possible antagonistic effect was more pronounced in the case of higher HT concentrations. The decrease in activity during storage coincided with the drastic reduction in the vitamin C content which took place during storage, due to the higher concentrations of HT added, which would partly confirm this antioxidant behavior.

However, it is important to note that all these tests were carried out on aqueous extracts of strawberry puree, in which all the phenols or vitamin C present in the strawberry itself may not be present. To try to resolve this possibility, another series of tests was carried out in which the strawberry puree had come directly into contact with the DPPH radical. These measurements of antioxidant activities were probably more representative of the total antioxidant activity existing in strawberry puree. In these experiments, it was observed that there was no clear antagonistic effect even in the case of high concentrations of HT (Figure 4c).

The possibility of synergistic, additive or antagonistic interactions using the DPPH assay was also evaluated using a combination index (CI) based on the method described by Chou and Talalay [26] using CompuSym software [27]. It was performed by directly suspending the strawberry puree samples, the HT solutions and the different strawberry-HT puree mixtures in the DPPH reagent. The results of these experiments are shown in Table 2. It can be seen how the CI values obtained are mostly bordering on the value 1, which would indicate an additive effect of the interaction. However, after 48 h of storage, some of the strawberry-HT puree combinations showed some synergistic effects (CI ˂ 1) and at 144 h they were mostly CI ˃ 1 values, moving away from the value 1, which would indicate an antagonistic effect.

The different antioxidant methods tested proved that the functionalization of strawberry puree containing HT significantly increased in vitro antioxidant activity. Although lower concentrations of HT regenerated the vitamin C content, as was demonstrated above, the presence of other antioxidants from strawberry puree or higher HT concentrations decreased the total amount of antioxidants, including vitamin C, but still improved the antioxidant activity overall.

## 3. Materials and Methods

### 3.1. Materials

HT was extracted and purified from olive by-products using a chromatographic system following the processes described by Fernández-Bolaños et al. [28,29]. Strawberries were purchased from a local market. Ascorbic acid (A.A), metaphosphoric acid, and acetic acid were purchased from Sigma-Aldrich (St. Louis, MO, USA)

### 3.2. Sample Preparation

#### 3.2.1. Strawberry Puree Sample Preparation

Strawberries were purchased from a local market during the months of April to May. All varieties (Calinda & Inspire) were produced in the community of Andalusia, Spain. The strawberries were homogenous in size, color and appearance, without signs of mechanical damage or fungal infection, and were manually selected for treatment. They were washed with water and allowed to dry on filter paper, then the chalices and peduncles were removed manually and the remaining fruit was crushed. They were homogenized and placed in duplicate glass bottles (60 g).

Mixing strawberry puree with HT was done by adding different concentrations of HT to 60 g of strawberry puree. Final concentrations of HT between 0.05–1 mg HT/g of strawberry puree were added. In addition, samples were prepared without adding HT (control). The samples were homogenized for 5 min and subsequently stored at 4 °C until analysis and throughout the test.

#### 3.2.2. Model System Solution of Ascorbic Acid (AA) with HT

A solution of 0.6 mg/mL A.A in water was prepared and HT was added for a final concentration between 0.05–1 mg/mL solution.

### 3.3. Extraction of Vitamin C in Strawberries

The method developed by Van de Velde et al. [30] was used for extraction of vitamin C from strawberry puree. Briefly, an extraction solution with metaphosphoric acid (30 g/L) and acetic acid (80 g/L) was used. 25 mL of extraction solution were added to 5 g of strawberry puree with and without HT. The mixture was homogenized for 1 min (Ultra-Turrax^®^ T50 Basic, IKA, Staufen, Germany) and centrifuged at 3000× *g* for 10 min at 4 °C. The supernatant was separated and placed in an ice bath until the determination of vitamin C.

### 3.4. Determination of Vitamin C (Total Acid Ascorbic)

The method developed by Stevens, et al. [31] was used to determine the vitamin C content in strawberry puree and the model system solution using ascorbic acid as a standard. Total ascorbate (ascorbic acid (AA) + dehydroascorbic acid (DHAA)) was measured with incubation with dithiothreitol (+DTT) reducing the oxidized form of vitamin C. Briefly:

Total Ascorbate Assay: Twenty microliters of sample or standard were distributed into two wells for the two repetitions. Twenty microliters of 5 mM DTT (+DTT) were added (in 0.4 M phosphate buffer, pH 7.4) for 20 min at 37 °C. Ten microliters of N-ethyl maleimide (NEM; 0.5% *w/v* in water) were added, mixed, and left for 1 min at room temperature. Eighty microliters of color reagent were added (solution A: 31% orthophosphoric acid, 4.6% *w/v* TCA, and 0.6% *w/v* iron chloride; solution B: 4% 2,2-dipyridyl (*w/v* made up in 70% ethanol). Solutions A and B were mixed 2.75 parts (A) to 1 part (B). The covered plate was incubated for 40 min at 37 °C. 

Ascorbate Assay: The same assay but without DTT (−DTT) allowed for the measurement of ascorbic acid alone.

The absorbance was read at 550 nm using a microplate reader. The amount of dehydroascorbic acid was obtained by comparing the sample with DTT and without DTT:

(+DTT) − (−DTT) = dehydroascorbic acid.

### 3.5. Determination of Hydroxytyrosol by HPLC-DAD

The HT was quantified using a Hewlett- Packard 1100 liquid chromatography system with a C-18 column (Teknokroma Tracer Extrasil ODS-2, 250 mm × 4.6 mm i.d., 5 μm). The system was equipped with a diode array detector (DAD; the wavelengths used for quantification were 254, 280, and 340 nm) and Rheodyne injection valves (20 μL loop). The mobile phases were 0.01% trichloroacetic acid in water (A) and acetonitrile (B) utilizing the following gradient over a total run time of 30 min: 95% A initially, 85% A at 15 min, 20% A at 20 min, 0% A at 23 min, 95% A at 27 min, and 95% A at 30 min, until the run was completed. Quantification was carried out by integration of peaks at 280 nm wavelength with reference to calibrations made using external standards. The linearity of the standards curve was expressed in terms of the determination coefficients plots of the integrated peak area against the concentration of the same standard. These equations were obtained over a wide concentration range in accordance with the levels of these compounds in the samples. The system was linear in all cases (r > 0.99). Three replicates on the same day were carried out.

### 3.6. Determination of the Total Phenolic Content

Total phenolic content was determined by the Folin−Ciocalteu spectrophotometric method and expressed as grams of gallic acid equivalents [32].

### 3.7. Antiradical Activity: 2,2-diphenyl-1-picrylhydrazyl (DPPH)

Free radical-scavenging capacity was measured using the DPPH method as described previously [33]. Briefly, 5 μL of each extract (extractions of strawberry puree in water), and 195 μL of the DPPH solution (3.8 mg/50 mL of DPPH solution in methanol) were placed in each microplate well in triplicate. For each sample, a blank with methanol instead of the DPPH solution was included. For the determination of the antiradical activity of the extracts, a microplate reader (550 model from Bio-Rad, Hercules, CA, USA) was used. After 30 min of reaction, the absorbance at 490 nm was measured. The assay was calibrated using 6-hydroxy 2,5,7,8-tetramethylchroman-2-carboxylic acid (Trolox), and the results were expressed in mg/mL TE (Trolox equivalent).

In the case of strawberry puree, antioxidant activity was evaluated as described by Fuentes-Alventosa et al. (2009) [34] with slight modifications. Briefly, 2–6 mg of strawberry puree was suspended in 7 mL of DPPH reagent (3.8 mg/50 mL methanol). After 30 min of continuous agitation, the material was centrifuged and the absorbance of the supernatant was measured at 490 nm. The results were expressed in terms of Trolox equivalent antioxidant capacity in mmol Eq Trolox/L of sample.

### 3.8. Ferric Reducing Antioxidant Power (FRAP)

The reducing power assay was performed according to the procedure described in a previous study [33]. Briefly, ten microliters of each extract and standard, and 10 µL of 6 mM FeCl_3_ in 5 mM citric acid were placed in each microplate well in quadruplicate. For each sample, a blank without FeCl_3_ was included. After dosification, the microplate was incubated for 20 min at 50 °C in an oven. Following this, 180 µL of 5 g/L 2-2, dipyridyl solution in 1.2% trichloroacetic acid was added to each well. Afterwards, a delay of 30 min was programmed in the reader before reading at 490 nm. The assay was calibrated using 6-hydroxy-2,5,7,8-tetramethylchroman-2-carboxylic acid (Trolox), and the results were expressed in mmolEq Trolox/L. 

### 3.9. CompuSyn Analysis

To determine the synergistic, additive or antagonistic effect of the antioxidant activity between HT and the strawberry puree, data were analyzed using CompuSyn software (CompuSyn Inc., Paramus, NJ, USA). This program is based on Chou-Talalay’s multiple drug effect equations [26], which defines the theoretical basis for the synergistic, additive and antagonistic effects between components in a mixture by the combination index (CI). CompuSyn software defines synergy as CI values less than 1, CI = 1 additive, and CI > 1 antagonism [28].

### 3.10. Statistical Analysis 

The results were expressed as mean values ± standard deviations. STATGRAPHICS^®^ plus software was used for the statistical analysis. Comparisons amongst samples were made using one-way analysis of variance (ANOVA) and the LSD method. A *p*-value < 0.05 was considered significant.

## 4. Conclusions

The present study shows that the natural antioxidant hydroxytyrosol at low concentrations (0.05 mg HT/g strawberry) stabilized vitamin C in strawberry puree during storage. The addition of hydroxytyrosol to a strawberry puree improved its antioxidant activity at low concentrations, since this activity was due to both vitamin C and HT contents. However, at high concentrations (1 mg HT/g strawberry), although the activity was improved due to the concentration of HT, the vitamin C content in the puree decreased dramatically, indicating the concentration of an antioxidant such as HT is very important when it comes to functionalizing a food such as a juice or fruit puree. An adequate concentration protects its main components and is a source of beneficial properties for health, while an inadequate concentration can affect some active components which are vital, as in the case of vitamin C.

## Figures and Tables

**Table 1:** Evolution of the concentration of hydroxytyrosol (HT) added to a model system solution of ascorbic acid during storage at room temperature (RT) and at 4 °C (**a**), and added to a strawberry puree during storage at 4 °C (**b**). The data are show as mean ± SD of duplicates. Letters (a–c), indicate significant differences of concentration among groups at *p* < 0.05.

(a)																	
	HT Loaded (mg/mL)	24 h				48 h				72 h				96 h			
		$\bar{x}$	±	SD		$\bar{x}$	±	SD		$\bar{x}$	±	SD		$\bar{x}$	±	SD	
RT	0.05	0.04	±	0.02	a	0.04	±	0.02	a	0.04	±	0.01	a	0.04	±	0.01	a
	0.10	0.09	±	0.00	a	0.09	±	0.00	a	0.08	±	0.00	a	0.08	±	0.00	a
	0.50	0.49	±	0.01	a	0.48	±	0.02	a	0.48	±	0.02	a	0.42	±	0.01	b
	1.00	0.99	±	0.01	a	0.98	±	0.00	a	0.95	±	0.01	b	0.88	±	0.02	c
4 °C	0.05	0.05	±	0.00	a	0.04	±	0.00	a	0.04	±	0.00	a	0.04	±	0.00	a
	0.10	0.11	±	0.01	a	0.09	±	0.00	a	0.09	±	0.01	a	0.09	±	0.00	a
	0.50	0.50	±	0.03	a	0.49	±	0.02	a	0.43	±	0.02	b	0.44	±	0.00	b
	1.00	0.92	±	0.05	a	0.86	±	0.01	b	0.93	±	0.03	a	0.88	±	0.01	b
**(b)**																	
**HT Loaded (mg/g Strawberry)**		**24 h**				**48 h**				**72 h**				**96 h**			
		$\bar{x}$	**±**	**SD**		$\bar{x}$	**±**	**SD**		$\bar{x}$	**±**	**SD**		$\bar{x}$	**±**	**SD**	
0.05		0.05	±	0.00	a	0.05	±	0.00	a	0.05	±	0.00	a	0.05	±	0.00	a
0.10		0.11	±	0.01	a	0.10	±	0.00	a	0.09	±	0.00	a	0.09	±	0.00	a
0.50		0.50	±	0.01	a	0.40	±	0.02	b	0.39	±	0.02	b	0.38	±	0.01	b
1.00		0.92	±	0.12	a	0.79	±	0.02	b	0.76	±	0.03	b	0.72	±	0.05	b

**Table 2:** Combination index (CI) of the antioxidant interactions of strawberry puree and hydroxytyrosol (HT) at different ratios and during storage at 4 °C by the DPPH method.

			CI		
mg HT/mg Strawberry	Strawberry (mg)	HT (mg)	24 h	48 h	144 h
0.25	30	7.5	1.216	0.854	0.853
0.25	40	10	1.039	0.891	1.112
0.25	50	12.5	1.146	0.915	1.362
0.5	10	5	1.916		0.702
0.5	20	10	0.758	0.917	1.260
0.5	30	15	1.025	1.034	1.060
0.5	40	20	0.950	1.069	1.565
1	10	10	1		
1	20	20	0.950	1.069	1.565
1	30	30	0.943	0.951	1.750

The DPPH assay was performed by directly suspending the samples of strawberry puree, HT solutions and the various strawberry-HT puree mixtures in the DPPH reagent. Combination index (CI) value by CompuSyn analysis: CI values between 0.95 and 0.85 suggest a slight synergy (green), values in the range of 0.95 and 1.15 suggest a near additive effect and values higher than 1.15 indicate antagonistic effect.
